# Genome sequences of 38 *Fusarium oxysporum* strains

**DOI:** 10.1186/s13104-022-06112-1

**Published:** 2022-06-27

**Authors:** Fatemeh Sabahi, Zia Banihashemi, Mara de Sain, Martijn Rep

**Affiliations:** 1 Department of Plant Protection, College of Agriculture, Shiraz University, Shiraz, Iran; 2 Molecular Plant Pathology, University of Amsterdam, Amsterdam, 1098 XH The Netherlands

**Keywords:** *Fusarium oxysporum* f. sp. *melonis*, Melon, Whole-genome sequencing

## Abstract

**Objective:**

Wilt caused by *Fusarium oxysporum* f. sp. *melonis* (Fom) is one of the most widespread and destructive melon diseases worldwide. Whole-genome sequencing data of a diverse set of Fom strains, as well as several non-pathogenic strains isolated from melon from different parts of the world are described here. These data shed light on the genetic diversity, population structure and the potential evolutionary trajectories which have led to the emergence of different Fom races, and will facilitate identification of avirulence genes which will be helpful to develop resistant melon cultivars.

**Data description:**

Genomic DNA was extracted from mycelium of 38 *Fusarium oxysporum* (Fo) strains collected from different parts of the world including Belgium, China, France, Iran, Israel, Japan, Mexico, New Zealand, Spain, the Netherlands, and the United States. The genomes were sequenced to ≈ 20 × coverage using the Illumina Hiseq Xten system, resulting in paired-end reads of 151 bp and assemblies of 1675 (Fom-18L) to 4472 (Fom-R12-13) scaffolds. The genome sequences are available in the National Center for Biotechnology Information (NCBI) and the Sequence Read Archive (SRA) under Project number PRJNA596396 and PRJNA596396, respectively. The presented data set can be useful to identify the genes associated with pathogenic strategies.

## Objective

Melon (*Cucumis melo* L.), whose total production in 2018 was more than 27 million tons worldwide, is one of the most cultivated horticultural food crops in subtropical and tropical regions, and is also grown widely in temperate zone countries [1, 2]. Melons originated in Africa or in Turkey and southwestern-Asia [3–6]. Like many other crops, melons are affected by numerous pathogens. Among these, the fungus *Fusarium oxysporum* f. sp. *melonis* (Fom), the causal agent of Fusarium wilt, is one of the most yield-limiting pathogens of melon worldwide [7]. Fom strains colonize the roots and enter the water-conducting xylem vessels, which leads to yellowing, wilting and eventually death of the infected plant. Two dominant resistance genes against Fom in musk melon, *Fom1* and *Fom2*, are currently in use [8]. Based on the resistance conferred by these two genes, Fom strains are classified into four races: races 0, 1, 2, and 1.2 [9]. Race 0 only causes disease in cultivars without *Fom1* and *Fom2*. Race 1 can infect *Fom1-*containing cultivars and race 2 is able to infect cultivars containing *Fom2*. Race 1.2 overcomes both resistance genes. Currently, nine vegetative compatibility groups (VCGs), VCGs 0130–0138, are identified in Fom strains worldwide [10–12]. These VCGs correspond to clonal lines [13]. Fusarium wilt is difficult to manage because the pathogen can persist in the soil as dormant propagules for decades and can also persist by colonizing crop residue or roots of non-susceptible crops grown in rotation with melon [14]. The use of resistant cultivars is the most effective way of controlling Fusarium wilt, and the nature of pathogen and diversity of virulence in the pathogen population is important for the success of the breeding program for melon Fusarium wilt resistance [2]. Therefore, the availability of complete whole-genome sequencing data of Fom strains representing the world-wide genetic variation and all four races is important, and will also provide an opportunity to identify avirulence genes [15], especially *AVRFom1*, which will be helpful to identify resistant musk melon cultivars. In addition, whole-genome sequences provide an essential data set to identify genetic markers for Fom (race) detection, to gain insight into the evolution of Fom races, and for research into the genetic basis of Fom pathogenicity. We carried out whole-genome sequencing of 38 *Fusarium oxysporum* (Fo) strains which includes three, five, eight, and thirteen strains of races 0, 1, 2, and 1.2, respectively, four strains of which we could not determine the race, and five non-pathogenic strains (Table 1). We have already used these data for comparative population genomics to identify putative effector genes and better understand the genetic relationships between the different Fom lineages [13].

**Table 1:** Overview of whole-genome sequence data files of *Fusarium oxysporum* f. sp. *melonis* strains

Label	Name of data file/data set	File type (file extension)	Data repository and identifier
Data Set 1	Whole-genome sequencing of *Fusarium oxysporum* strains that cause *Fusarium* wilt on melon	No file type	PRJNA59639 https://identifiers.org/ncbi/bioproject:PRJNA596396 [21]
Data file 1	Fom-FomGol	FASTQ (fq.gz)	NCBI SRA Database https://identifers.org/ncbi/insdc.sra:SRR11348735.1 [22]
Data file 2	Fom-Busherh-2 s	FASTQ (fq.gz)	NCBI SRA Database https://identifers.org/ncbi/insdc.sra:SRR11348736.1 [23]
Data file 3	Fom-660A-17	FASTQ (fq.gz)	NCBI SRA Database https://identifers.org/ncbi/insdc.sra:SRR11348737.1 [24]
Data file 4	Fom-18L	FASTQ (fq.gz)	NCBI SRA Databasehttps://identifers.org/ncbi/insdc.sra:SRR11348738.1 [25]
Data file 5	Fom047	FASTQ (fq.gz)	NCBI SRA Database https://identifers.org/ncbi/insdc.sra:SRR11348741.1 [26]
Data file 6	Fom043	FASTQ (fq.gz)	NCBI SRA Database https://identifers.org/ncbi/insdc.sra:SRR11348742.1 [27]
Data file 7	Fom026	FASTQ (fq.gz)	NCBI SRA Databasehttps://identifers.org/ncbi/insdc.sra:SRR11348743.1 [28]
Data file 8	Fom025	FASTQ (fq.gz)	NCBI SRA Database https://identifers.org/ncbi/insdc.sra:SRR11348744.1 [29]
Data file 9	Fom024	FASTQ (fq.gz)	NCBI SRA Database https://identifers.org/ncbi/insdc.sra:SRR11348745.1 [30]
Data file 10	Fom023	FASTQ (fq.gz)	NCBI SRA Database https://identifers.org/ncbi/insdc.sra:SRR11348746.1 [31]
Data file 11	Fom021	FASTQ (fq.gz)	NCBI SRA Database https://identifers.org/ncbi/insdc.sra:SRR11348747.1 [32]
Data file 12	Fom020	FASTQ (fq.gz)	NCBI SRA Database https://identifers.org/ncbi/insdc.sra:SRR11348748.1 [33]
Data file 13	Fom017	FASTQ (fq.gz)	NCBI SRA Database https://identifers.org/ncbi/insdc.sra:SRR11348750.1 [34]
Data file 14	Fom015	FASTQ (fq.gz)	NCBI SRA Database https://identifers.org/ncbi/insdc.sra: SRR11348751.1 [35]
Data file 15	Fom014	FASTQ (fq.gz)	NCBI SRA Database https://identifers.org/ncbi/insdc.sra:SRR11348752.1 [36]
Data file 16	Fom007	FASTQ (fq.gz)	NCBI SRA Database https://identifers.org/ncbi/insdc.sra:SRR11348753.1 [37]
Data file 17	Fom-Yazd2	FASTQ (fq.gz)	NCBI SRA Database https://identifers.org/ncbi/insdc.sra:SRR11348754.1 [38]
Data file 18	Fom-Taip2a	FASTQ (fq.gz)	NCBI SRA Database https://identifers.org/ncbi/insdc.sra:SRR11348755.1 [39]
Data file 19	Fom-Tai3	FASTQ (fq.gz)	NCBI SRA Database https://identifers.org/ncbi/insdc.sra:SRR11348756.1 [40]
Data file 20	Fom-T61-1	FASTQ (fq.gz)	NCBI SRA Database https://identifers.org/ncbi/insdc.sra:SRR11348757.1 [41]
Data file 21	Fom-Seif3a	FASTQ (fq.gz)	NCBI SRA Database https://identifers.org/ncbi/insdc.sra:SRR11348758.1 [42]
Data file 22	Fom-R12-13	FASTQ (fq.gz)	NCBI SRA Database https://identifers.org/ncbi/insdc.sra:SRR11348759.1 [43]
Data file 23	Fom-Pathtah	FASTQ (fq.gz)	NCBI SRA Database https://identifers.org/ncbi/insdc.sra:SRR11348760.1 [44]
Data file 24	Fom-P13	FASTQ (fq.gz)	NCBI SRA Database https://identifers.org/ncbi/insdc.sra:SRR11348761.1 [45]
Data file 25	Fom-NYFom62	FASTQ (fq.gz)	NCBI SRA Database https://identifers.org/ncbi/insdc.sra:SRR11348762.1 [46]
Data file 26	Fom-NYFom3	FASTQ (fq.gz)	NCBI SRA Database https://identifers.org/ncbi/insdc.sra:SRR11348763.1 [47]
Data file 27	Fom-Nasr1	FASTQ (fq.gz)	NCBI SRA Database https://identifers.org/ncbi/insdc.sra:SRR11348764.1 [48]
Data file 28	Fom-Mah9a	FASTQ (fq.gz)	NCBI SRA Database https://identifers.org/ncbi/insdc.sra:SRR11348765.1 [49]
Data file 29	Fom-KT2a	FASTQ (fq.gz)	NCBI SRA Databasehttps://identifers.org/ncbi/insdc.sra:SRR11348766.1 [50]
Data file 30	Fom-Khaf1	FASTQ (fq.gz)	NCBI SRA Database https://identifers.org/ncbi/insdc.sra:SRR11348767.1 [51]
Data file 31	Fom-I1-1	FASTQ (fq.gz)	NCBI SRA Database https://identifers.org/ncbi/insdc.sra:SRR11348769.1 [52]
Data file 32	Fom-I-17	FASTQ (fq.gz)	NCBI SRA Database https://identifers.org/ncbi/insdc.sra:SRR11348770.1 [53]
Data file 33	Fom-Kavar-22	FASTQ (fq.gz)	NCBI SRA Database https://identifers.org/ncbi/insdc.sra:SRR11348768.1 [54]
Data file 34	Fo-nonpath5	FASTQ (fq.gz)	NCBI SRA Database https://identifers.org/ncbi/insdc.sra:SRR11348739.1 [55]
Data file 35	Fo-nonpath2	FASTQ (fq.gz)	NCBI SRA Database https://identifers.org/ncbi/insdc.sra:SRR11348740.1 [56]
Data file 36	Fo-nonpath-TO1	FASTQ (fq.gz)	NCBI SRA Database https://identifers.org/ncbi/insdc.sra:SRR11348749.1 [57]
Data file 37	Fo-nonpath-2Ma4-5	FASTQ (fq.gz)	NCBI SRA Database https://identifers.org/ncbi/insdc.sra:SRR11348771.1 [58]
Data file 38	F-nonpath-Barmshour	FASTQ (fq.gz)	NCBI SRA Database https://identifers.org/ncbi/insdc.sra:SRR11348772.1 [59]

### Data description

In order to obtain insight into the genomic diversity and relationship between clonal linages of Fom, we collected a diverse set of Fom strains originating from different geographical locations across the world including Belgium [33], China [31, 32], France [24, 35, 36, 43], Iran [22, 23, 38–40, 42, 44, 45, 48–51, 53–59], Israel [52], Japan [30, 41], New Zealand [28, 29], Spain [37], the Netherlands [26], and the United States [25, 34, 46, 47]. Genomic DNA was isolated as described in Sabahi et al. [13]. The quality of extracted genomic DNA was evaluated by Nano-Drop, Qubit, and agarose gel electrophoresis. Library preparation was performed by the Hartwig Medical Foundation and by the RNA Biology and Applied Bio-informatics department at the University of Amsterdam. Illumina sequencing (151 bp paired-end) was performed on a Hiseq Xten system at the Hartwig Medical Foundation (Amsterdam, the Netherlands). Raw reads were trimmed to remove low-quality bases and adapter sequences using Trimmomatic (v0.39) [16]. Quality control of both the raw and trimmed sequence reads was done by FastQ Screen (v0.14.0) and FastQC (v0.11.3). Genomic sequences were assembled using CLC genomics workbench v8.5 with Default CLC settings, except “minimum contig length = 500” which resulted in 1675 (Fom-18L) to 4472 (Fom-R12-13) scaffolds. The smallest and largest de novo assembly have a size of 51.0 megabasepairs (Mbp) (Fom024) and 56.5 Mbp (F-nonpath-Barmshour), respectively. The N50 scaffold size, calculated to evaluate the quality of the assembly, ranged from 99,974 bp (Fom-R12-13) to 527,362 bp (Fo-nonpath-TO1). Prediction of putative effector genes in Fom genome sequences was carried out by searching uninterrupted ORFs in regions of 2500 bp or 5000 bp downstream of a miniature impala (mimp) terminal inverted repeat (TIR) [17]. In the present data set, 40 new candidate effector genes were identified [13]. Phylogenetic analysis of Fom strains was performed by searching for homologues of 440 conserved Fol4287 genes in the sequences of the Fom genomes using megaBLAST with default parameters. From this, 422 genes which had a single hit that overlaps > 70% with the query sequences and showed at least 80% identity to the query were selected to continue the analysis. We used MUSCLE [18] to construct a sequence alignment for each query and a custom python script to concatenate these alignments (https://github.com/marads/conserved_gene_tree_scripts). Concatenated alignments were trimmed by trimAl [19]. We used RaxML v8.2.12 to generate the phylogenetic tree with 100 bootstrap replicates [20]. According to the presence/absence of candidate effector genes and phylogenetic analyses based on 422 conserved genes, all Fom strains used in our study were grouped in nine genetic lineages [13]. The presence of highly similar effector patterns between some distant lineages are suggestive of horizontal chromosome transfer between these lineages [13]. The data presented herein will provide useful resources to identify new avirulence genes which are important for being able to assess the efficiency and the durability of resistance genes in agricultural settings, and also contributes to understanding evolutionary trajectories that have led to the emergence of races in Fom.

## Limitations

The number of Fom strains collected and sequenced limits analysis of genomic variation in the population. Our data do not include Fom strains belonging to VCGs 0137 and 0138. It should be noted also that we obtained short-reads with a mean coverage of about 20 × which might not be suitable for some genomic analyses.

## Data Availability

All sequence data described herein have been deposited in DDBJ, ENA, and NCBI database as the Sequence Read Archive (SRA) format (https://www.ncbi.nlm.nih.gov/sra?linkname=bioproject_sra_all&from_uid=596396) under the project number PRJNA596396 [21]. Please see Table 1 and the references [22–59] for details and links to the data.

## Abbreviations

DDBJ: DNA data bank of japan
ENA: European nucleotide archive
F: *Fusarium*
Fo: *Fusarium* *oxysporum*
Fom: *Fusarium* *oxysporum* f. sp. *melonis*
NCBI: National center for biotechnology information
Nonpath: Non-pathogen
SRA: Sequence read archive
VCG: Vegetative compatibility group
